# Correction to “Evaluation of Thresholding Methods for Activation Likelihood Estimation Meta‐Analysis via Large‐Scale Simulations”

**DOI:** 10.1002/hbm.70387

**Published:** 2025-11-13

Frahm, L., E. C. Cieslik, F. Hoffstaedter, et al. 2022. “Evaluation of Thresholding Methods for Activation Likelihood Estimation Meta‐Analysis via Large‐Scale Simulations.” *Human Brain Mapping* 43, no. 13: 3987–3997. https://doi.org/10.1002/hbm.25898.

There is a mistake in the author contributions of the paper. The text reads “Lennart F Edna C. Cieslik, and Robert Langner came up with the first drafts of the paper.” This should have read: “Lennart Frahm came up with the first draft of the paper. Edna C. Cieslik and Robert Langner gave feedback.”

We apologize for this error.

